# Pulp Revascularization of Immature Permanent Teeth: A Review of the Literature and a Proposal of a New Clinical Protocol

**DOI:** 10.1155/2014/737503

**Published:** 2014-10-14

**Authors:** Mélanie Namour, Stephanie Theys

**Affiliations:** Department of Pediatric Dentistry, Catholic University of Louvain, Belgium

## Abstract

Tissue engineering is a growing field. In the near future, it will probably be possible to generate a complete vital tooth from a single stem cell. Pulp revascularization is dependent on the ability of residual pulp and apical and periodontal stem cells to differentiate. These cells have the ability to generate a highly vascularized and a conjunctive rich living tissue. This one is able to colonize the available pulp space. Revascularization is a new treatment method for immature necrotic permanent teeth. Up to now, apexification procedures were applied for these teeth, using calcium dihydroxide or MTA to produce an artificial apical barrier. However, the pulp revascularization allows the stimulation of the apical development and the root maturation of immature teeth. Two pulp revascularization techniques are used in the literature, one using calcium dihydroxide and the second using a triple antibiotic paste. Based on these two different pulp revascularization protocols, which obtain the desired therapeutic success, the literature will be reviewed and analyzed according to the relevance of their choice of materials. Based on the literature, we propose a new relevant protocol and a new mixture of antibiotics.

## 1. Introduction

Tissue engineering is a growing field. In the near future, it will probably be possible to generate a complete vital tooth from a single stem cell. Stem cells are in fact totipotent cells, which have the capacity to proliferate and to produce cells, which are capable of differentiating into specialized cells.

Two types of stem cells exist: embryonic stem cells and adult stem cells (or postnatal cells) [1]. Concerning pulp revascularization, mature stem cells are rather of interest. These cells are found in many sites of the dental element: in the pulp, in the apical papilla, and in the periodontal ligament [1, 2]. These clonogenic cells, rapidly differentiating, have the capacity of inducing dentin-pulp regeneration if differentiating into appropriate cells. In addition, the pulp, which is a product from migration of the neural crest, would probably be a very good candidate to allow nerve regeneration [1]. Regarding the daily practice, it is imperative to find ways to save as much as possible the vitality of stem cells from the dental element and induce their differentiation.

Pulp revascularization is dependent on the ability of residual pulp and apical and periodontal stem cells to differentiate [3–5]. These cells have the ability to generate a highly vascularized and a conjunctive rich living tissue. This one is able to colonize the available pulp space. Subsequently, these stem cells will differentiate into newly formed odontoblasts that will induce an apposition of hard tissue. The nature of this latter is unknown yet [1].

Revascularization is a new treatment method for immature necrotic permanent teeth. Indeed, it would provide, after treatment, a vital tooth that would be able to complete its root maturation. Up to now, apexification procedures were applied for these teeth:

- using calcium dihydroxide to induce the formation of an apical calcified barrier;
- using mineral trioxide aggregate (MTA) to produce an artificial apical barrier.

Both methods have shown to be effective regarding the narrowing of the apical foramen of an immature tooth. However, the pulp revascularization allows also the stimulation of the apical development and the root maturation of immature teeth (root growth and thickening of dentinal walls and natural apexification).

Indications for treatment of pulp revascularization are the presence of deep caries or trauma inducing a stop in the development of root canal of an immature tooth. It is important to keep in mind that an endodontic treatment on an immature tooth, often necessary up to now, involves a root canal treatment on an open apex tooth with thin and fragile walls. This will involve the persistence of a weakened tooth with often a reserved long-term prognosis due to the remaining of an intrinsic fragility and to the difficulty to obtain a good sealing of an open apex. Revascularization technique would allow the growth of root and thus avoiding the remaining of thin and fragile walls. It will reduce the risk of root fracture [6]. This is not the case with apexification treatment.

Immature teeth with a large open apex and short roots seem to be more conducive to the successful treatment of pulp revascularization.

A great importance is given to maintaining the vitality of a tooth in order to keep a possibility of “alert” signal in case of pathogenic stimuli. Losing its innervation and vascularization, a tooth is more vulnerable to any lesion. The maintaining of dental vitality allows better defenses in case of future possible infections.

This pulp revascularization is used for necrotic immature permanent teeth. Even if pulp has lost its vitality, residual pulp stem cells are able to survive. Apical papilla stem cells can also survive to an apical lesion thanks to an abundant blood supply [1, 6–8].

## 2. Operative Protocol

Two pulp revascularization techniques are found in the literature: one using calcium dihydroxide (Table 1) and another using a triple antibiotic paste (Table 2) for disinfection of pulp necrosis. Both are two-step procedure.

Second step takes place two or three weeks after the first one, only if the tooth is asymptomatic and if there is a visual reducing of the apical lesion.

In pulp revascularization, at three months postoperative, the tooth is normally asymptomatic and about nine months later X-ray radiography shows an increasing thickness of dentinal walls and an apical closure. Root development and apical closure may be visible after three months.

Based on these two different pulp revascularization protocols, which obtain the desired therapeutic success according to their authors, the literature is reviewed and analyzed according to the relevance of their choice of materials. The objective is to define a protocol that would seem to be the most adapted.

## 3. Discussion

The success of pulp revascularization treatment depends on three elements: root canal disinfection, the presence of a scaffold (blood clot), and hermetic coronary filling [2].

The generation of a functional tissue requires three key elements: stem cells, growth factors, and a scaffold [9].

It is known that the quality of root canal restoration is questionable when residual bacteria are present in the canal. This one could proliferate and eventually induce a reinfection. Therefore, it is essential to have an immune system of quality, major canal disinfection, and a coronary and apical filling allowing no recontamination.

The first part of discussion will be concerned with instrumentation in root canal. Then, the discussion will mention points of divergence between both protocols: irrigation, disinfection, and pulp-capping material. Finally, tissue obtained after pulp revascularization treatment will be mentioned.

### 3.1. Instrumentation

Most of authors agree to advocate no instrumentation procedure. Using root canal instrument could not only increase fragility of dentin walls but also injure stem cells present in the apical area of these dentin walls. These also contain growth factor imprisoned during dentinogenesis. Growth factor and other cells essential for the regeneration process could also be eliminated by instrumentation. Two types of cells are required to achieve a normal root development: odontoblasts and epithelial cells of Hertwig's sheath. These two cell types are present in abundance in the apical area of immature teeth and are able to resist inflammation phenomena [1, 6–8]. These cells will be able to differentiate into secondary odontoblasts that will generate dentin on root canal walls [1] and thus allow root maturation. No instrumentation procedure remains consistent with vital stem cells preservation and avoids weakening of already thin root canal walls [4, 10, 11].

According to the study of Cehreli et al. [12], even if the number of cases is not sufficient to be statistically significant, it can be noticed that some patients have regained tooth sensitivity (vitality) after treatment. That was observed only in no instrumentation treatment cases.

Thus, elements mentioned so far in favor of no instrumentation protocol seem to be more advised.

### 3.2. Irrigation

Irrigators play a role of primary disinfection. They should have a maximal bactericidal and bacteriostatic effect while having a minimal cytotoxic effect on stem cells and fibroblasts to allow their survival and ability to proliferate.

Pulp infection can usually spread to the apical region and create a canal acidic environment. This one is not conducive to the creation of tissue regeneration. Bacterial invasion of root canal system causes the formation of bacterial biofilms. Those hang on root canal walls, entrance of dentinal tubules, and in the apical area containing more complex anatomical crevices. At these locations, bacterial biofilms are more resistant to disinfection procedures. Bacteria existing in depth and within the biofilm are in lag phase and therefore refractory to action of antibiotics and irrigators. To ensure optimal root canal disinfection for tissue regeneration, it is necessary to disrupt or eliminate biofilms. Using a tool such as fine “interdental brush” could probably be useful to disrupt biofilms without injuring hard dental tissue. However, the disadvantage of this kind of tool is the potential risk of leaving nonbiocompatible residues (hairbrush) into root canal.

Activating the irrigation solution within the root canal system is the only possibility to realize disintegration of the bacterial biofilm in noninstrumented areas. It justifies the use of endosonics means. They generate a process of cavitation that induces a temperature increase of the irrigator and currents propelling the irrigator in all crevices. The whole have the effect of potentiating the efficacy of irrigator in order to disintegrate bacterial biofilm [13]. However, during this activation, it is essential to avoid touching the canal walls with endosonic tool in order to respect the decision to avoid any contact between dentinal walls and instruments.

*(A) Hydrogen Peroxide*. Solvent properties of hydrogen peroxide are almost nonexistent, but it has an interesting hemostatic action. Hydrogen peroxide is antiseptic by release of oxygen radical. Unfortunately, his action is too short and quickly neutralized by organic debris. Moreover, it requires a rinse to reduce pain and possible postoperative gaseous emphysema.

*(B) Chlorhexidine*. Chlorhexidine 2% gel was proposed as a temporary medication. It has good action on candida and gram^+^ bacteria by the carryover effect. Indeed, its positively charged molecules confer the property of being adsorbed by the dentin walls and thus allow release of chlorhexidine for at least two to twelve weeks, preventing reinfection of the root canal during this period [14]. Despite this advantage, chlorhexidine does not have an effective dissolving action.

*(C) Sodium Hypochlorite*. So far, sodium hypochlorite remains irrigator reference in endodontic. It has a solvent action on necrotic tissue and an antiseptic effect widely demonstrated [15]. However, it must be supplemented by a desalting. Recommended concentrations vary between 0.5% and 5.25% [16–19]. Cytotoxicity of sodium hypochlorite is proportional to its concentration. The concentration of 2.5% seems to be the best compromise between efficiency and lack of toxicity [20]. Furthermore, Cunningham showed that elevation of the temperature at 37°C of the 2.5% sodium hypochlorite solution potentiates its solvent power and its efficiency becomes comparable to that of the solution to 5, 25% [21].

*(D) Iodine*. Iodine is bactericide, antifungal, antiviral, sporicidal, and sedative. Purulent secretions and blood do not inactivate it [22]. Its disadvantage is that it colors dental tissues in brown [23].

*(E) Ethylene Diamine Tetraacetic Acid (EDTA) + Irrigators*. Chelators are weak acids, which react with the mineral portion of dentinal walls. They replace calcium ions with sodium ions, which combine with the dentin to give soluble salts. EDTA-type chelating allows better wettability of the irrigator and a removal of the smear layer [24, 25].

According to Trevino who studies effects of irrigants on the survival of human stem cells of the apical papilla, the use of EDTA before irrigators would allow maximum survival of these cells [26]. 17% of EDTA is often used in cases of bacterial infection to remove the smear layer and allow access to the entrance of dentin tubules (allowing a better chance of joining tissue of regeneration) and induce a better penetration of the irrigator (increases wettability of the irrigator) and of root canal medications [24, 25]. EDTA is also a “sealer” that maximizes bacteriostatic and bactericidal effects of different agents. Its chelating effect would allow the release of growth factors imprisoned in the dentin during dentinogenesis. That would stimulate the proliferation of stem cells [27, 28]. Since EDTA appear to have many advantages, it is important to know how to combine the irrigators. Ring et al. have compared effects of chlorhexidine and hypochlorite after treatment with EDTA [29]. They show that there is no survival stem cell after using a combination of EDTA and 2% chlorhexidine. Moreover, precipitates chlorhexidine salts are formed and maintained in root canal. These precipitates can be toxic and prevent cell adhesion to the canal wall. The combination of EDTA and 6% of hypochlorite seems to moderately reduce vitality of stem cells. It is also recommended to rinse with saline after irrigating in order to minimize the risk of possible precipitates and to remove residual debris and remain of irrigant [26].

### 3.3. Disinfection

*(A) Calcium Dihydroxide*. Calcium dihydroxide, Ca(OH)_2_, is a strong base (pH = 12.5–12.8); its ionic dissociation in Ca^2+^ and OH^−^ induced genesis of hard tissue (apexification, tertiary dentin) and has an antibacterial effect by the release of ion OH^−^ [30]. These ions OH^−^ damage the cytoplasmic membrane, suppress the bacterial enzyme activity, denature proteins, damage DNA and thus inhibit any replication, and inactivate endotoxins. However, it seems that they have no power over biofilms [31].

Calcium dihydroxide has a low coefficient of dissociation (0.17), which is a good clinical feature since it allows a long-term release of Ca^2+^ and OH^−^. Seven days seem sufficient to reduce the bacterial load in root canal at a level of negative culture [32].

According to Nosrat et al. [6], it appears that the basic pH of calcium dihydroxide denatures proteins and could induce necrosis of apical tissue. In any way, it allows thickness increasing of dentinal walls [6, 33].

However, it seems that the dentine (consisting of hydroxyapatite), residues of pulp necrosis, and inflammatory exudates decrease its antibacterial power. For this reason, its effectiveness of disinfection is discussed for in vivo application [34]. Calcium dihydroxide would not be effective on* Enterococcus faecalis*. Acids bacterial products and phosphates from hydroxyapatite of the dentin that limit the diffusion of ions H^+^ and OH^−^ rapidly neutralize its pH [34].

According to some research, Ca(OH)_2_ would increase the expression of some kind of kinases (extracellular signals by phosphorylation), which are indicators of proliferation of stem cells from pulp and ligament [35]. Therefore, used in usual concentrations, it would not be cytotoxic for stem cells and would support their proliferation [36]. However, tricalcium silicates cements, such as MTA, Ca(OH)_2_, or Biodentine, have a weakening effect on dentin because of their pH [4, 5]. These damages would be repairable over time but only for MTA and Biodentine [33].

A study realized on cells' cultures showed the direct effect of intracanal medications on stem cells from apical papilla. Calcium dihydroxide used at a concentration of 0.01 mg/mL for canal disinfection allows survival of 100% of the apical stem cells. Even at higher concentration, 1 mg/mL, Ca(OH)_2_ would also give a maximal survival of stem cells. At the same concentration, antibiotics paste only allows between 33% and 56% cells survival. Used in normal concentrations, antibiotics paste is more toxic than Ca(OH)_2_, unless if they are used in appropriate concentrations (lower concentrations) [36].

*(B) Triple Antibiotic Paste (TAP)*. According to Chuensombat et al. [37] who studies in vitro antibacterial efficacy and cytotoxic effects of a triple antibiotic paste, it appears that an antibiotic used alone is less cytotoxic than the use of a mixture of antibiotics. To eliminate bacteria belonging to the spectrum of an antibiotic, this one should be used at a minimum concentration of 25 *μ*g/mL. No antibiotics have a spectrum large enough to be active against all types of bacteria present in root canals and apical regions; a combination of antibiotics is essential to cover a maximum range of action. Antibiotics pastes must be used in proper concentration for a balance between a lower cytotoxicity against stem cells (cytotoxicity increases with dose) and a maximum bacterial disinfection. An in vitro study has shown that a TAP concentration of 39 *μ*g/mL would be best for application in disinfection root canal [37].

Hoshino and Takushige showed [38] that mixture paste of three antibiotics with propylene glycol put into root canal with a Lentulo and at a concentration of 20 *μ*g/mL decreases by more than 99%; the average number of bacterial colonies is present [38]. Another in vitro study conducted by Hoshino et al. shows that each antibiotic used alone is ineffective against bacteria present in pulp, dentine, and apical lesions, while the trio of antibiotics allows complete sterilization of germs [38, 39]. Sato et al. developed triple antibiotic paste [40]. Expected to cover at best different root canal bacteria, the three antibiotics consisting of the paste are minocycline (spectrum of gram^+^ and gram^−^), ciprofloxacin (spectrum of gram^+^ and gram^−^), and metronidazole (spectrum of anaerobic bacteria and protozoa) [37].

Acid pH of minocycline is not favorable to cultivation of stem cells; it would probably facilitate cell permeability of the antibiotic, which would keep long-term cytotoxicity. Ciprofloxacin has also an acid ph. Metronidazole is the only antibiotic of the mixture to have a neutral pH and thus it has no cytotoxicity for needed stem cells [37].

The triple antibiotic paste seems to be biocompatible but its current problem is the possible bacterial resistance.

Minocycline is a semisynthetic tetracycline derivative with a similar action spectrum. It may be replaced by cefaclor in order to avoid any risk of unaesthetic coronary coloring [41] because minocycline binds to ions Ca^++^ by chelation and form insoluble complexes [42]. However, cefaclor appears to be less effective against enterococci. An alternative could be to previously seal the dentinal tubules of the pulp chamber (etching and bonding) [4].

Tetracycline would have ability to inhibit collagenase and metalloproteinases; it is not cytotoxic and is capable of increasing the level of interleukin-10 (anti-inflammatory cytokine). Replacing minocycline by cefaclor due to coronary coloring, we will not be deprived of the benefits of this tétracycline dérivative? Should we not rather choose directly the option of sealing dentin tubules to avoid coronary discoloration? [4].

Metronidazole and ciprofloxacin could induce the formation of fibroblasts [4].

According to Bose et al., the use of triple antibiotic paste shows the highest percentage increase in thickness of the dentinal canal walls compared to the two other intracanal medications (calcium dihydroxide and formocresol) [43].

*Enterococcus faecalis* is a bacterium of the most importance because it is present in infection resistant to apical treatments [44]. Current enteric bacterium, gram-positive, can survive and grow in dental root canal without requiring the presence of other bacteria. This bacterium has the ability to invade and survive easily in the dentinal tubules. According to Adl et al., antibiotics have a better action against* Enterococcus faecalis* than calcium dihydroxide. Indeed, the triple antibiotic powder (metronidazole, ciprofloxacin, and minocycline) combined with a saline solution shows the lowest minimum inhibitory concentration (MIC) against* Enterococcus faecalis* (MIC = 77.5 *μ*g/mL). The second place is for a combination of triple antibiotic paste and 2% chlorhexidine with similar results than a combination of minocycline and saline (MIC = 325 mg/mL). The least effective group is combination of calcium dihydroxide and chlorhexidine (MIC = 195 000 *μ*g/mL). Calcium dihydroxide combined with saline is absolutely not effective against* Enterococcus faecalis*. Triple antibiotic paste is very effective against bacteria often present in apical lesion and minocycline seems to be its most active component. However,* Enterococcus faecalis* is not a prevalent bacterium in primary infection of permanent immature teeth [44].* E. faecalis* is also present in the endocarditis, based on the antibiotic treatment for this disease; we can imagine using similar components for pulp regeneration. Furthermore, their bactericidal spectrum is similar to that commonly used for root disinfection [45, 46].

Pinheiro et al. [47] reported twenty isolated kinds of bacteria in filled root canal with persistent apical lesion. It appears that these highly resistant bacteria are apparently sensitive to tetracycline and doxycycline [47].

Since the choice would seem to lead to pulp revascularization using antibiotic paste, it is important to find antibiotics with neutral ph. Indeed, this would be a favorable environment for stem cells differentiation. Moreover, physical properties of dentin walls could be affected by leaving an acid component for long term in root canal. Therefore, the best will be antibiotics with neutral pH and covering spectrum of minocycline and ciprofloxacin.

An alternative could be the use of a chloramphenicol solution stabilized at neutral ph. Chloramphenicol is used in the absence of alternatives for the local treatment of conjunctivitis, keratitis, and corneal ulcers. Unfortunately, it seems little used due to the risk of adverse effects that must be taken into account.

Other combinations of antibiotics used for infective endocarditis [46] may be considered for their cellular tolerance and their neutral pH. The use of ampicillin (active on bacteria gram^+^ and gram^−^) combined with gentamycin (active on bacteria gram^−^) was proposed. On the other hand, a recent study [46] proposed the following combination for its safety and efficiency ampicillin with ceftriaxone (cephalosporin, third generation). Therefore, we can propose the same association combined with metronidazole for anaerobic germs.

A second proposition can be done. The combination of metronidazole, penicillin G [45], and streptomycin [48] (efficient against gram^−^ as* E. Coli* and gram^+^ as* Staphylococcus aureus*). A third proposition may be metronidazole, ceftriaxone, and amikacin [49] (gram^−^).

### 3.4. Pulp-Capping Materials (MTA and Biodentine)

After disinfection step, a suitable scaffold to encourage generation of new tissue must fill the root canal. At the same time, coronary access must be sealed to prevent further reinfection [50].

Before discussing the possibilities of capping root canal, an issue arises. Induction of the root canal bleeding is done to bring in situ fibrin, platelets, and growth factor. All these elements are indispensable to formation of tissue regeneration. It would also create a matrix from which the growth of new vital tissue is possible into root canal space. The question is could previously prepared platelet rich fibrin (PRF) be included in root canal during bleeding [26]. This would contribute to bring more growth factors and to create a biological tissue scaffold, which promotes tissue growth (reduction of waiting time in comparison with time required for the formation of coagulum).

In vitro studies have demonstrated that calcium dihydroxide and MTA, with their high pH, exert a severe weakening effect on dentin walls during a period of two weeks to two months [51]. However, samples sealed with MTA seem to recover their mechanical properties as fracture toughness after one year. It is not the case with calcium dihydroxide [33].

Biodentine has the same mechanical characteristics as human dentin. Moreover, upon application of this material in a cavity, it seems to fully expand and fill the space by its plasticity [52]. Another advantage is absence of coloring the cervical area unlike MTA, excepted using white MTA.

### 3.5. The Tissue Regeneration

Claus et al. [53] and Ritter et al. [15] described histological tissue regeneration in animals. They described the existence of a significant neovascularization and the presence of connective cells [15, 53]. Through studies on animal cuts, the apposition material-inducing thickening of root walls may be of different nature dentin, cementum, or even bone [54]. Therefore, this procedure is not a process of pulp revascularization but a process of tissue regeneration. The inability to obtain sections of human teeth after revascularization is a handicap for understanding and validating this process. Only radiographic assessments of in vivo clinical studies and the use of a laser quantifying blood flow (laser Doppler flowmetry) can give us an idea of treatment success [15, 55]. Testing vitality with cold also seems to be a good indicator of success.

Through the analysis of articles in the literature, a new protocol could be proposed (Table 3).

## 4. Conclusion

Following the analysis of pulp revascularization approaches discussed so far, before opening the tooth, it seems effective to isolate the tooth with a rubber dam and disinfect it with 10% povidone iodine (iso-Betadine) to maximal reduce of oral bacterial concentration.

After opening of the pulp chamber, no root canal instrumentation is still recommended to avoid altering dentinal walls and stem cells present on their surfaces. However, the use of a breast nerve may be useful to remove majority of the infected and necrotic pulp without damaging the root walls of immature teeth.

Primary irrigation with EDTA combined with 6% sodium hypochlorite seems to be the best solution as EDTA (little cytotoxic and opening dentin tubules) allows better penetration of the irrigants (and medications) in root canal crevices and tubules. A release of growth factors imprisoned during dentinogenesis could be expected. Sodium hypochlorite remains irrigator base for root canal disinfection. If using a 2.5% sodium hypochlorite concentration, its effectiveness and its solvent power may be potentiated by warming at 37°C. It also seems that rinsing with saline could only bring a benefit to treatment.

Regarding root canal temporary medication, triple antibiotic paste used has good concentration that seems to be the most appropriate in order to avoid any problems associated with calcium dihydroxide (weakening dentinal walls, inducing tissue necrosis, and decreasing effectiveness by infectious exudates). Indeed, the three antibiotics cover at best action spectra of root canal bacteria and show minimum stem cells cytotoxicity when used in adequate concentration (0.39 *μ*g/mL).

During the second step of the procedure, the addition of PRF in root canal may be beneficial. PRF provides an additional supply of blood components, such as growth factors and a more solid support (scaffold) allowing growth of the generated tissue.

Biodentine would be proposed for root canal capping because it appears to have the necessary assets for this procedure (same mechanical properties as human dentine expand to entirely fill space by its plasticity that would increase crown-root tightness, absence of cervical area coloration, and very low cytotoxicity).

For the final hermetic filling, the choice of material does not greatly matter but it should be as airtight as possible and sustainable.

## Figures and Tables

**Table 1a:** (a)

First step	
Local anesthesia	
Isolation of the tooth with a rubber dam	
Opening of the pulp chamber to canal entrance (pulpotomy)	
Irrigation of root canal (often with 10 mL sodium hypochlorite at 2.5%)^a^	
*No instrumentation in root canal *	
Preparation of calcium hydroxide paste^b^	
Insertion of the paste in the pulp chamber and in the coronary part (third or half) of root canal (with a cotton ball)	
Sealing of the access cavity with a temporary filling	

^a^According to authors, nature and concentration of the irrigator can vary.

^b^Ca(OH)_2_-sterile water in a 3 : 1 ratio.

**Table 1b:** (b)

Second step	
(two or three weeks later if asymptomatic tooth and/or absence of fistula)	
Local anesthesia without vasoconstrictor^a^	
Isolation of the tooth with a rubber dam	
Opening the tooth to have a access to root canal	
Removal of the calcium hydroxide paste	
Copious irrigation of root canal with sodium hypochlorite	
Rinsing root canal with sterile water	
Drying root canal with paper cones	
An apical bleeding is caused by irritation of the apical region with a 15 K-file lime^b^	
Preparation of mineral trioxide aggregate (MTA) and its placement on the clot in order to form a hermetic sealing	
Place a wet a cotton ball on MTA filling	
Sealing of the cavity with a temporary filling	

^a^In order to not inhibit the future apical bleeding.

^b^It takes 15 minutes to obtain a blood clot. If a root canal is not bleeding, it is possible to transfer blood from one root canal to another. Blood level must be at least 2-3 mm below the cement-enamel junction.

**Table 2a:** (a)

First step	
Local anesthesia	
Isolation of the tooth with a rubber dam	
Disinfection of the tooth with 10% povidone-iodine (iso-Betadine) before opening it^a^	
Opening of the pulp chamber to canal entrance (pulpotomy)	
Irrigation of root canal^b^ with 20 mL sodium hypochlorite (1.25%–5.25%) then with physiological serum and finally with 2% chlorhexidine	
*No instrumentation in root canal *	
Drying root canal with paper cones	
Insertion of the triple antibiotic paste^c^ into root canal	
Place a cotton ball at the root canal entrance	
Sealing of the access cavity with a temporary filling	

^a^According to the authors, disinfection is done or not.

^b^According to the authors, irrigation may vary.

^c^Mixture of equal proportion of three antibiotics: metronidazole, ciprofloxacin, and minocycline bonded with propylene glycol. Minocycline may be replaced by cefaclor to avoid inducing coloration.

**Table 2b:** (b)

Second step	
(two or three weeks later if asymptomatic tooth and/or absence of fistula)	
Local anesthesia without vasoconstrictor^a^	
Isolation of the tooth with a rubber dam	
Disinfection of the tooth with 10% povidone-iodine (iso-Betadine) before opening it^b^	
Opening the tooth to have a access to root canal	
Removal of the triple antibiotic paste using irrigation with sodium hypochlorite (1.25%–5.25%) then with physiological serum and finally with 2% chlorhexidine^c^	
An apical bleeding is caused. Blood level must be at the cement-enamel junction.	
Preparation of mineral trioxide aggregate (MTA) and its placement on the clot^d^ in order to form a hermetic sealing	
Place a wet a cotton ball on MTA filling	
Sealing of the cavity with a temporary filling	

^a^In order to not inhibit the future apical bleeding.

^b^According to the authors, disinfection is done or not.

^c^Irrigation is done in order to make space for the future blood clot.

^d^It takes 15 minutes to obtain a blood clot.

**Table 3a:** (a)

First step	
Local anesthesia	
Isolation of the tooth with a rubber dam	
Disinfection of the tooth with 10% povidone-iodine (iso-Betadine) before opening it	
Opening of the pulp chamber to canal entrance (pulpotomy)	
Application of Biodentine on dentinal tubules of the pulp chamber^a^	
Root canal disinfection with 17% EDTA following by 2.5% sodium hypochlorite warming at 37°C	
Drying root canal with paper cones	
Insertion of the triple antibiotic paste^b^ into root canal with a Lentulo^c^	
Place a cotton ball at the root canal entrance	
Sealing of the access cavity with a temporary filling	

^a^It is important to keep root canal entrance accessible. This action is intended to seal dentin tubules in order to avoid any subsequent medicine staining.

^b^Mixture of equal proportion of three antibiotics: metronidazole, ciprofloxacin, and minocycline bonded with propylene glycol (concentration of 0.39 *μ*g/mL).

^c^Without overflow at the pulp chamber to avoid any future staining.

**Table 3b:** (b)

Second step	
(two weeks later if asymptomatic tooth and/or absence of fistula)	
Local anesthesia without vasoconstrictor^a^	
Isolation of the tooth with a rubber dam	
Disinfection of the tooth with 10% povidone-iodine (iso-Betadine) before opening it^b^	
Opening the tooth to have a access to root canal	
Removal of the triple antibiotic paste using irrigation with 2.5% sodium hypochlorite then with physiological serum	
An apical bleeding is caused. Blood level must be at the cement-enamel junction After filling root canal with blood, previously prepared PRF can be add	
Twelve minutes later, application of Biodentine on the clot formed around PRF in order to close access to root canal	
Final hermetic filling after hardening of Biodentine	

^a^In order to not inhibit the future apical bleeding.
